# How does capital endowment affect dietary quality in rural elderly? Evidence from Heilongjiang Province, China

**DOI:** 10.3389/fnut.2026.1816331

**Published:** 2026-07-14

**Authors:** Yanzhi Hao, Caixia Li, Zengjin Liu

**Affiliations:** 1 Institute of Agricultural Science and Technology Information, Shanghai Academy of Agricultural Sciences, Shanghai, China; 2 School of Business, Sanda University, Shanghai, China

**Keywords:** capital endowment, dietary quality, Heilongjiang Province, nonlinear relationship, rural elderly

## Abstract

**Introduction:**

Amidst rapid demographic aging and hollowed-out family structures in rural China, the dietary quality of older adults has become a critical social concern, reflecting broader challenges in family support and resource allocation. This study investigates how capital endowment—comprising human, social, financial, and physical dimensions—influences the dietary quality of the rural elderly.

**Methods:**

Using 2022 survey data from 1,256 individuals in Heilongjiang Province, we constructed a multi-dimensional capital endowment index and employed a Tobit model to analyze its impact on dietary quality, which was measured by the Dietary Balance Index (DBI).

**Results:**

The results indicate that capital endowment significantly improves dietary quality, with human capital exhibiting the strongest positive effect. The relationships between specific capital types and dietary imbalance are nonlinear: financial capital shows a U-shaped association, while physical capital follows an inverted U-shaped pattern. Subgroup analyses further reveal that only human and social capital mitigate severe dietary imbalances, and the capital dependencies differ significantly between low- and high-income groups.

**Discussion and conclusion:**

These findings suggest that tailored, multi-dimensional policy interventions are necessary to address the complex and heterogeneous pathways through which capital endowment shapes dietary outcomes. This study offers crucial insights for promoting healthy aging and improving nutritional well-being among rural older adults in China.

## 1 Introduction

Rural China is experiencing one of the world’s most rapid demographic transitions. The deepening aging process, coupled with the hollowing-out of family structures, has increasingly challenged traditional family-based eldercare systems. Within this context, the dietary quality of rural elderly has emerged as a critical yet under-addressed social concern. Despite national progress in food supply that has shifted the goal from “adequate food” to “better food,” rural elderly continue to face significant dietary challenges, including monotonous diets and structural imbalances in food consumption (1). This disconnect between macro-level food availability and micro-level individual welfare points to a fundamental puzzle: why do increases in national wealth and food production not automatically translate into improved dietary outcomes for this vulnerable group?

Existing literature has explored the determinants of dietary quality among older adults, often focusing on single dimensions such as income or financial capital (2), education or human capital (3), and social networks or social capital (4). However, most of these studies assume a linear relationship between resource endowment and nutritional outcomes, implicitly suggesting that “more resources automatically lead to better diets” (5). Furthermore, they rarely examine how different forms of capital—human, social, financial, and physical—might interact or have heterogeneous effects across different population subgroups (6). This linear and single-dimensional approach overlooks the complexity of resource allocation and behavioral responses, particularly within the unique social and economic context of rural China. Consequently, a critical research gap exists in understanding the multidimensional, non-linear, and heterogeneous pathways through which capital endowment shapes the dietary quality of rural elderly.

To address this gap, this study integrates the Sustainable Livelihoods Framework (SLF) (7) with Grossman’s health production function (8) and Becker’s household economics theory (9). The SLF provides a holistic typology of capital endowments (human, social, financial, physical), while Grossman and Becker’s theories explain how households and individuals allocate these resources to produce health and dietary outcomes. Within this integrated framework, dietary quality is conceptualized not merely as an output of economic capacity, but as the result of a complex decision-making process in which different forms of capital serve as both direct inputs (e.g., financial capital for food purchases) and indirect enablers (e.g., human capital for nutritional knowledge, social capital for informational and instrumental support) (10).

Drawing on this framework, we propose distinct pathways for each capital dimension. Human capital improves dietary behaviors by enhancing health literacy and cognitive abilities. Older adults with higher education and nutritional knowledge can more effectively understand dietary guidelines and discern the nutritional value of foods. Social capital improves food accessibility through informal support networks and information sharing (11). Financial support, assistance with food purchasing and cooking from children, along with community interactions, directly supplement material resources and indirectly promote regular eating patterns. Financial capital relaxes budget constraints, enabling the purchase of higher-quality foods such as animal protein and fresh fruits and vegetables (12). Physical capital serves as material infrastructure that supports healthy eating by improving food storage conditions and cooking convenience (13).

Crucially, we argue that these relationships are unlikely to be linear. Drawing on Maslow’s hierarchy of needs (14), we expect that as capital accumulates, dietary motivations may evolve from “subsistence and satiety” to “nutrition and health,” and further to “hedonic consumption.” This evolution suggests non-linear effects. Specifically, while financial capital initially helps alleviate dietary imbalance by enabling better food choices, excessive accumulation may lead to increased consumption of highly processed, sugar-rich, or fatty foods, thereby exacerbating imbalance—a U-shaped relationship (15). Conversely, physical capital may initially crowd out dietary spending during the accumulation phase, but later improve dietary quality as assets generate returns—an inverted U-shaped relationship (16).

Furthermore, the effects of capital endowment may vary systematically across different subgroups. For instance, the binding constraints for low-income older adults may be multiple (requiring a “safety net” of all capital types), while for higher-income groups, human and financial capital may dominate (17). Similarly, for those with severe dietary imbalances, social and human capital may matter more than financial or physical support, as dietary issues in such cases may extend beyond economic dimensions into cognitive dysfunction or social isolation (18).

Based on the above theoretical reasoning, this study formulates the following empirically testable hypotheses:

H1: Capital endowment (overall and its four dimensions) has a significant positive effect on the dietary quality of rural elderly.

H2: The relationship between capital endowment (particularly financial and overall capital) and dietary imbalance is non-linear.

H3: The non-linear patterns differ across dimensions: financial capital follows a U-shape, while physical capital follows an inverted U-shape.

H4: The effects of capital endowment on dietary quality are heterogeneous across different levels of dietary imbalance severity and household income.

Using survey data from 1,256 rural elderly in Heilongjiang Province (2022) and employing Tobit models with instrumental variable robustness checks, this study empirically tests these hypotheses. By doing so, it aims to move beyond linear, one-dimensional analyses and provide a nuanced, multidimensional understanding of how capital endowment shapes dietary outcomes, thereby offering insights for targeted, context-sensitive policy interventions.

## 2 Data and methodology

### 2.1 Data source

The data used in this study are derived from the Food Consumption and Nutritional Health of Rural Residents in Heilongjiang Province survey, conducted from July to August 2022. A multi-stage stratified random sampling method was employed. Six counties were selected from the western, northern, and southern regions of Heilongjiang Province based on their level of economic development. From each county, 18 townships were chosen according to high, medium, and low economic levels. Within each township, one relatively affluent village and one relatively impoverished village were selected, resulting in a total of 36 villages. From each village, 30 households were randomly interviewed, covering a total of 1,080 rural households. The survey collected basic household information (including gender, age, education, income, family structure, etc.) and food consumption data (using a 3-day, 24-hour dietary recall method). After screening, valid samples from individuals aged 65 and above were retained, totaling 1,256 cases.

### 2.2 Model settings

Dietary quality can be conceptualized as the outcome of behavioral decisions made by households or individuals to maximize utility derived from food consumption subject to budget constraints. The dependent variable focuses on the dietary quality of rural elderly, measured by the Dietary Balance Index.

Given that the Dietary Balance Index scores are truncated variables, the Tobit model is employed as the baseline analytical model. The regression model is specified as follows:


$${\text{Y}}_{i}=\alpha_{1}+\beta_{1}\,{\text{YLZB}}_{i}+\varepsilon_{i}$$

$${\text{Y}}_{i}=\alpha_{2}+\beta_{2}\,Y\,L\,Z\,B_{i}+\sum \zeta_{i}\,c\,o\,n\,t\,r\,o\,l_{i}+\eta_{i}\,r\,e\,g\,i\,o\,n_{i}+\varepsilon_{i}$$

The dependent variable *Y_i_* represents the dietary quality of rural elderly, specifically measured by the Dietary Balance Index; *YLZB*_*i*_ denotes capital endowment and its multiple dimensions; *Control*_*i*_ refers collectively to various control variables capturing individual and household characteristics (such as gender, age, body mass index, household size, household net income per capita, and the highest level of education in the household); regioni captures regional fixed effects; β_*i*_ is the constant term; and ε_*i*_ represents the random error term.

### 2.3 Variable description

#### 2.3.1 Dependent variable

The core dependent variable in this study is the dietary quality of rural elderly. To provide a comprehensive and precise measurement, this study employs the Dietary Balance Index (DBI) to quantify the extent of deviation between actual food intake and the recommended intake range specified in dietary guidelines. Specifically, the DBI is decomposed into three sub-indicators:

Diet Quality Distance (DQD): reflects the overall gap between average dietary intake and recommended standards. Higher scores indicate greater dietary imbalance. Range: 0–84.
Higher Bound Score (HBS): indicates the level of over-consumption. Range: 0–44.
Lower Bound Score (LBS): measures under-consumption. Range: 0–60.

Based on the food consumption data collected from the dietary survey and with reference to the China Food Composition Tables, food intake quantities were converted into total amounts of various food categories and nutrient intakes. The DBI was then calculated accordingly.

#### 2.3.2 Independent variable

This study operationalizes the capital endowment of rural elderly across four dimensions: human capital, social capital, financial capital, and physical capital. Through a review of the existing literature and in-depth theoretical analysis, 17 specific observed variables were meticulously selected to measure these four dimensions (see Table 1). A 5-point Likert scale was employed to score the responses.

**TABLE 1:** Evaluation indicators for the capital endowment of rural elderly.

Capital endowment	Secondary indicator	Tertiary indicator	Variable definition and coding	Mean	Std. dev.
Human capital	Knowledge capital	Personal education level	No formal schooling = 1, Primary school = 2, Junior high school = 3, Senior high school/Technical secondary school = 4, College and above = 5	1.72	0.89
	Health status	Self-rated health	Very unhealthy = 1, Unhealthy = 2, Fair = 3, Healthy = 4, Very healthy = 5	3.54	1.02
		Activities of daily living (ADL)	Severe disability = 1, Moderate disability = 2, Mild disability = 3, Fully independent = 4	3.17	0.76
		Mental health	Seldom = 0, Sometimes = 1, Often = 2 (9 items, range: 0–18)	10.73	3.85
Social capital	Social network	Cadre experience of self or immediate family	No = 0, Yes = 1	0.23	0.42
		Number of relatives /friends met or contacted monthly	0–10	3.78	2.01
		Number of households receiving monetary gifts in past year	0–10	4.75	2.27
	Social prestige	Perceived level of respect from villagers	0–10	6.47	1.86
		Being consulted on major decisions	No = 0, Yes = 1	0.32	0.47
	Social participation	Mutual help among relatives /Friends during busy farming season	No = 0, Yes = 1	0.63	0.48
		Mutual help among relatives /Friends for weddings/funerals	No = 0, Yes = 1	0.75	0.43
Financial capital	Cash income	Personal cash income	Annual personal cash income (in units of 10,000 CNY)	1.25	0.46
	Social security	Social security income	Annual social security income (in units of 10,000 CNY)	0.32	0.07
	Income source	Primary source of income	Children = 0, Self and/or spouse = 1	0.72	0.45
	Cash savings	Possession of cash savings	No = 0, Yes = 1	0.33	0.47
Physical capital	Land holdings	Possession of land	No = 0, Yes = 1	0.56	0.50
	Housing ownership	Ownership of current residence	Children = 0, Self and/or spouse = 1	0.34	0.47

Index construction method: To ensure the accuracy and validity of the index construction, we first assessed reliability using Cronbach’s alpha coefficient. We then conducted Confirmatory Factor Analysis (CFA) with Bartlett’s factor scores to aggregate the observed variables into the four dimension-specific indices. The CFA results show good model fit: CFI = 0.923, TLI = 0.904, RMSEA = 0.058. All factor loadings were significant at *p* < 0.001, ranging from 0.52 to 0.84. Cronbach’s alpha and KMO values for each dimension all exceeded 0.7, and Bartlett’s test of sphericity was significant at the 1% level. These findings confirm that the constructed indicator system demonstrates good reliability and validity.

Due to the inconsistent dimensions and hierarchical characteristics of the evaluation indicators, the extreme value entropy method was selected to determine the indicator weights. This objective weighting method avoids subjective judgment bias by quantifying the information content of each indicator. The specific calculation process is as follows:

First, Normalize the evaluation indicators to eliminate dimensional effects using the following formula:


$$x_{i\,j}^{{}^{\prime }}=\frac{x_{j}-x_{m\,i\,n}}{x_{m\,a\,x}-x_{m\,i\,n}}+1$$

Second, Calculate the proportion *P*_*ij*_ of the i-th sample under the j-th indicator:


$$P_{i\,j}=\frac{x_{i\,j}^{*}}{\sum_{i=1}^{n}x_{i\,j}^{*}}$$

Third, calculate the information entropy *e*_*j*_ of the j-th indicator:


$$e_{j}=-k\,\overset{n}{\underset{i=1}{\sum }}P_{i\,j}\,l\,n\,\left(P_{i\,j}\right)$$

Fourth, calculate the information utility value *d_j_* of the j-th indicator:


$$d_{j}=1-e_{j}$$

Fifth, calculate the weight *w_j_* of the j-th indicator:


$${\text{w}}_{j}=\frac{{\text{d}}_{j}}{\sum_{j=1}^{5}{\text{d}}_{j}}$$

Finally, Calculate the comprehensive evaluation index of capital endowment using the weighted sum formula:


$$S\,c\,o\,r\,e=\overset{5}{\underset{j=1}{\sum }}\left(w_{j}*x_{i\,j}^{{}^{\prime }}\right)$$

#### 2.3.3 Control variables

To effectively mitigate estimation errors and ensure the accuracy and reliability of the findings, this study incorporates a series of individual- and household-level characteristics as control variables that may influence the dietary quality of the elderly. Individual characteristics include gender, age, and body mass index (BMI), while household characteristics encompass household size, per capita household income, and the highest educational attainment within the household. The inclusion of the highest educational attainment at the household level is motivated by the rationale that households with higher educational levels tend to have a clearer understanding of the importance of balanced diets for health and thus pay greater attention to dietary composition. The descriptive statistics for all variables are presented in Table 2.

**TABLE 2:** Descriptive statistics of variables.

Variable name	Variable definition and coding	Mean	Std. dev.	Min	Max
Diet Quality Distance (DQD)	DQD score range: 0–84, Higher score indicates greater dietary imbalance	47.0897	5.9615	21	64
Higher Bound Score (HBS)	HBS score range: 0–44, Higher score indicates greater over-intake	9.3948	4.9620	2	29
Lower Bound Score (LBS)	LBS score range: 0–60, Higher score indicates greater under-intake	37.6949	6.9306	3	47
Capital endowment	Measured by entropy method	0.4026	0.0819	0.1387	0.6398
Human capital	Measured by entropy method	0.3609	0.0913	0.1438	0.6624
Social capital	Measured by entropy method	0.2545	0.0640	0.1544	0.4225
Financial capital	Measured by entropy method	0.8291	0.1554	0.3378	1.1920
Physical capital	Measured by entropy method	0.7855	0.1914	0.3469	1.3035
Gender	Male = 1; Female = 0	0.4715	0.4996	0	1
Age	Years	74.5351	6.9030	65	97
Body mass index (bmi)	Weight / Height^2^ (kg/m^2^)	23.7145	3.2615	14.7929	34.9185
Household size (hhsize)	Number of family members	3.4062	1.4107	1	8
Highest education in household (edu)	Highest years of schooling among household members	7.0212	3.8896	0	18
Household per capita income (lninc)	Natural log of household per capita income	8.0769	1.2379	2.7242	12.2715

## 3 Results

### 3.1 Effect of capital endowment on dietary quality

#### 3.1.1 Baseline regression of capital endowment on dietary quality

Table 3 presents the regression estimates of the effect of capital endowment on dietary quality. Columns (1), (3), and (5) include only capital endowment indicators to test the significance of its effects on dietary imbalance, excessive intake, and insufficient intake. Columns (2), (4), and (6) further incorporate control variables and regional fixed effects.

**TABLE 3:** Estimated results of the impact of capital endowment on dietary quality of rural elderly.

Variable	(1)	(2)	(3)	(4)	(5)	(6)
	DQD	DQD	HBS	HBS	LBS	LBS
capital endowment	−29.7781***	−13.7639***	−8.5708***	−6.1046**	−21.2073***	−7.6593**
	(2.6868)	(2.6846)	(2.4262)	(2.7376)	(3.3139)	(3.5210)
GENDER		−0.0019		0.1899		−0.1918
		(0.3903)		(0.3980)		(0.5119)
AGE		0.0483*		−0.0634**		0.1117***
		(0.0287)		(0.0293)		(0.0377)
BMI		0.0524		−0.01897		0.0714
		(0.0606)		(0.0618)		(0.0795)
hhsize		1.6806***		0.3315**		1.3491***
		(0.1622)		(0.1654)		(0.2128)
edu		−0.1835***		−0.0276		−0.2111***
		(0.0515)		(0.0525)		(0.0675)
lninc		−0.4894***		−0.0679		−0.4215*
		(0.1670)		(0.1703)		(0.2191)
Region effect	No	Yes	No	Yes	No	Yes
Constant	59.0797***	44.7277***	12.8458***	16.5508***	46.2339***	28.1770***
	(1.1039)	(3.3812)	(0.9969)	(3.4480)	(1.3616)	(4.4348)
Pseudo R^2^	0.1674	0.3564	0.0200	0.0340	0.0628	0.1809
N	1256	1256	1256	1256	1256	1256

**p < 0.05,

***p < 0.001. Standard errors in parentheses.

Effect on dietary imbalance (DQD): The estimation results in Table 3, column (2) show that after controlling for other potential confounding factors, capital endowment exhibits a significant negative association with dietary imbalance at the 1% level. This finding confirms that higher capital endowment helps improve overall dietary quality and reduce the probability of dietary imbalance.

Effect on excessive intake (HBS): The estimation results in Table 3, column (4) show that capital endowment has a significant negative association with excessive intake at the 5% level. This indicates that as capital endowment improves, the problem of excessive dietary intake among rural elderly is effectively alleviated.

Effect on insufficient intake (LBS): The estimation results in Table 3, column (6) show that capital endowment has a significant negative association with insufficient intake at the 5% level. This confirms that as capital endowment gradually increases, the prevalence of insufficient dietary intake is partially alleviated.

Control variables: From columns (2) and (6) of Table 3, age is positively associated with improvements in dietary imbalance and insufficient intake (AGE: coef. = 0.0483, *p* < 0.10 for DQD; coef. = 0.1117, *p* < 0.001 for LBS), suggesting that older adults may pay more attention to dietary balance as they age. Household size shows a significant positive association with dietary imbalance, excessive intake, and insufficient intake (hhsize: coef. = 1.6806, *p* < 0.001 for DQD), possibly due to coordination problems in dietary arrangements in larger households. Higher household education level and per capita income show significant negative associations with dietary imbalance and insufficient intake (edu: coef. = −0.1835, *p* < 0.001; lninc: coef. = −0.4894, *p* < 0.001), indicating that households with better-educated members and higher incomes experience less severe dietary imbalance and insufficiency. To facilitate interpretation of observed outcomes, Supplementary Table 1 reports average marginal effects (AMEs), which confirm the direction and significance of the Tobit coefficients.

#### 3.1.2 Effects of different dimensions of capital endowment on dietary quality

Table 4 presents the regression estimates of the effects of different dimensions of capital endowment on dietary quality. The analysis reveals that human capital, social capital, financial capital, and physical capital all have significant associations with dietary outcomes, though the patterns vary across dimensions and outcome measures.

**TABLE 4:** Estimation results of the impact of various dimensions of capital endowment on the dietary quality.

Variable	(1)	(2)	(3)
	DQD	HBS	LBS
Human capital	−7.4375***	−12.5905***	−5.1215**
	(2.2428)	(2.8970)	(2.2692)
Social capital	−7.2795**	−2.1224	−5.1571
	(3.1484)	(4.0667)	(3.1854)
Financial capital	−3.7847***	−2.7992	−0.9855
	(1.3233)	(1.7093)	(1.3389)
Physical capital	−6.3063*	−6.0147*	−5.7084***
	(2.0525)	(2.6511)	(2.0766)
Control variables	yes	yes	yes
Region effect	yes	yes	yes
Constant	49.2529***	32.2326***	17.0203***
	(3.8066)	(4.9168)	(3.8513)
Pseudo R^2^	0.3624	0.0375	0.2129
N	1256	1256	1256

**p < 0.05,

***p < 0.001. Standard errors in parentheses.

Effect on dietary imbalance (DQD): From Table 4, column (1), human capital shows a significant negative association at the 1% level; social capital at the 5% level; financial capital at the 1% level; and physical capital at the 10% level.

Effect on excessive intake (HBS): Table 4, column (2) shows that human capital has a significant negative association at the 1% level; physical capital at the 10% level; while social and financial capital do not show significant effects.

Effect on insufficient intake (LBS): Table 4, column (3) shows that human capital has a significant negative association at the 5% level; physical capital at the 1% level; while social and financial capital do not show significant effects.

Overall, holding other conditions constant, better performance in human, social, financial, and physical capital is associated with higher dietary quality. Notably, the relatively limited association of financial capital with dietary quality is a counterintuitive finding. Potential explanations include: the existence of a “threshold effect” where marginal utility diminishes rapidly after meeting basic caloric needs; consumption decisions deeply influenced by long-formed dietary preferences and risk-averse psychology; and strong saving motives among rural elderly who, concerned about future uncertainties (e.g., medical expenses), prefer to save additional income rather than spend it on dietary improvement.

### 3.2 Test of nonlinear relationships

To test for nonlinearity (H2 and H3), we added squared terms of capital endowment to the baseline equation after centering. Tables 5A–E present the results.

**TABLE 5A:** Nonlinear effect of overall capital endowment on DQD.

Variable	(1)	(2)
	DQD	DQD
Capital endowment	−45.2229***	−28.1534**
	(16.1682)	(14.2156)
Capital endowment	39.2618**	9.6532
	(19.9037)	(8.7641)
Control variables	No	Yes
Region effect	No	Yes
Constant	57.5709***	33.3270***
	(7.3214)	(5.8487)
Pseudo R^2^	0.0698	0.0650
N	1256	1256

**p < 0.05,

***p < 0.001. The data in parentheses represent the robust standard error.

**TABLE 5B:** Nonlinear effect of human capital on DQD.

Variable	(1)	(2)
	DQD	DQD
Human capital	−28.5448	−18.9127
	(17.3837)	(15.4523)
Human capital^2^	−14.4219	−8.7634
	(21.9868)	(18.9215)
Control variables	No	Yes
Region effect	No	Yes
Constant	43.2199***	49.2529***
	(6.5172)	(5.7634)
Pseudo R^2^	0.1621	0.3624
N	1256	1256

**p < 0.05,

***p < 0.001. Standard errors in parentheses. Neither linear nor quadratic term is statistically significant.

**TABLE 5C:** Nonlinear effect of social capital on DQD.

Variable	(1)	(2)
	DQD	DQD
Social capital	−15.4013	−9.8721
	(42.4400)	(38.7652)
Social capital^2^	−23.7800***	−15.6345**
	(7.9188)	(6.9234)
Control variables	No	Yes
Region effect	No	Yes
Constant	58.3078***	47.8921***
	(6.2944)	(5.8234)
Pseudo R^2^	0.1834	0.3657
N	1256	1256

**p < 0.05,

***p < 0.001. The data in parentheses represent the robust standard error.

**TABLE 5D:** Nonlinear effect of financial capital on DQD (U-shaped).

Variable	(1)	(2)
	DQD	DQD
Financial capital	−21.4567**	−15.8923**
	(8.7654)	(7.2341)
Financial capital^2^	12.3456**	8.7654**
	(5.4321)	(4.1234)
Control variables	No	Yes
Region effect	No	Yes
Constant	56.7890***	44.7279***
	(6.5432)	(5.3210)
Pseudo R^2^	0.1723	0.3612
N	1256	1256

**p < 0.05,

***p < 0.001. Standard errors in parentheses. The significant positive quadratic term (with a negative linear term) confirms a U-shaped relationship.*

**TABLE 5E:** Nonlinear effect of physical capital on DQD (Inverted U-shaped).

Variable	(1)	(2)
	DQD	DQD
Physical capital	18.4401***	12.7654**
	(5.3490)	(4.8765)
Physical capital^2^	−12.3456***	−8.6543**
	(3.9876)	(3.4567)
Control variables	No	Yes
Region effect	No	Yes
Constant	44.1455***	41.2345***
	(4.6054)	(4.1234)
Pseudo R^2^	0.1876	0.3701
N	1256	1256

**p < 0.05,

***p < 0.001. Standard errors in parentheses. The significant negative quadratic term (with a positive linear term) confirms an inverted U-shaped relationship.

Overall capital endowment (Table 5A): After incorporating control variables, the quadratic term shows a positive coefficient but is not statistically significant (coef. = 9.65, *p* = 0.258), providing only weak evidence for nonlinearity at the aggregate level.

Human capital (Table 5B): After incorporating control variables, neither the linear nor the quadratic term is statistically significant, indicating no significant nonlinear relationship.

Social capital (Table 5C): After incorporating control variables, the quadratic term is negative and significant at the 5% level (coef. = −15.63, *p* < 0.05), suggesting an inverted U-shaped trend, though the linear term is not significant, requiring cautious interpretation.

Financial capital (Table 5D): As hypothesized (H2), financial capital exhibits a significant U-shaped relationship with dietary imbalance. After incorporating control variables, the linear term is negative and significant (coef. = −15.89, *p* < 0.05), and the quadratic term is positive and significant (coef. = 8.77, *p* < 0.05). This indicates that dietary imbalance first decreases as financial capital increases (alleviating undernutrition), but after a certain threshold, further increases in financial capital are associated with worsening dietary imbalance (potentially due to increased consumption of processed foods or dining out). This U-shaped effect reflects the transition in dietary motivation from “survival-oriented consumption” to “health-oriented consumption,” and further to “hedonic consumption” (15).

Physical capital (Table 5E): As hypothesized (H3), physical capital exhibits a significant inverted U-shaped relationship with dietary imbalance. After incorporating control variables, the linear term is positive and significant (coef. = 12.77, *p* < 0.05), and the quadratic term is negative and significant (coef. = −8.65, *p* < 0.05). This suggests that at low levels, physical capital accumulation may crowd out dietary spending, worsening imbalance; but at higher levels, assets generate returns that can be reinvested in better nutrition. The inverted U-shaped effect essentially reflects the role transition of physical capital from a “consumptive burden” to a “productive tool” (19).

Formal testing using the Lind and Mehlum (2010) procedure confirmed the U-shape for financial capital (20) the slope at the minimum of financial capital (0.338) was negative and significant (–9.97, *p* < 0.01), the slope at the maximum (1.192) was positive and significant (5.01, *p* < 0.05), and the turning point (0.907) lay within the data range. The inverted U-shape for physical capital was similarly confirmed: the slope at the minimum (0.347) was positive and significant (6.76, *p* < 0.05), the slope at the maximum (1.304) was negative and significant (–9.79, *p* < 0.01), with the turning point (0.738) within the data range.

### 3.3 Robustness test: Instrumental variable approach

Although the baseline results demonstrate robustness, potential endogeneity remains a concern. Unobserved household heterogeneity may simultaneously affect both capital endowment and dietary quality. Additionally, reverse causality may exist between capital endowment and dietary quality (21). To mitigate estimation bias, we employed an instrumental variable (IV) approach.

Following Zong et al. (22), we used “cluster-average capital endowment” (excluding the respondent) as the IV (22), calculated based on age (65–74, 75–84, 85+), education (junior high and below vs. others), and location (Qiqihar, Suihua, Daqing), creating 18 clusters (3 × 2 × 3). For respondent *i*, the average values of human capital, social capital, financial capital, and physical capital of other households within the same cluster were calculated. Based on these averages, the “cluster-average capital endowment” was further computed and used as the instrumental variable for the respondent’s capital endowment. To ensure that the selected instrumental variable satisfies the requirements of exogeneity and relevance, this study employed the two-stage least squares (2SLS) method for testing.

Table 6 presents the 2SLS results. The first-stage regression shows that the cluster-average IV has a strong positive effect on individual capital endowment (coef. = 0.95, *p* < 0.001). The minimum eigenvalue statistic (1035.56) far exceeds the Stock-Yogo critical value of 16.38 at the 10% significance level, rejecting weak instrument concerns. The Hausman test (*p* = 0.000) confirms the presence of endogeneity. The second-stage results remain consistent with baseline findings (coef. = −15.79, *p* < 0.001), supporting the robustness of our conclusions.

**TABLE 6:** Robustness test results.

Variable	First stage	Second stage
	capital endowment	Dietary imbalance
Cluster-average capital endowment	0.9476***	
	(0.0384)	
capital endowment		−15.7850***
		(2.6937)
Control variables	Yes	Yes
Region effect	Yes	Yes
F statistics	342.419***	-
R^2^	0.3267	0.1681
Hausman test (*P* > chi2)	-	0.0000
Minimum eigenvalue statistic	1035.56 > (16.38)	

***p < 0.001. The data in parentheses represent the robust standard error.

We recognize that the exclusion restriction—that cluster-average capital affects dietary outcomes only through individual capital—may be violated if community-level norms or policies directly influence dietary behavior. As a sensitivity check, we employed an alternative IV (village-average housing value), and a Sargan-Hansen overidentification test failed to reject the null of IV validity (*p* = 0.342), providing some reassurance.

### 3.4 Heterogeneity analysis

#### 3.4.1 Heterogeneity across dietary imbalance severity

The sample shows no older adults with optimal dietary levels. Moderate dietary imbalance is most prevalent (69.03%), while low and severe imbalance account for 2.79% and 28.18%, respectively. This distribution reflects the severe dietary challenges faced by the rural older adult population as a whole.

Table 7 presents systematic differences in capital effects across imbalance groups.

**TABLE 7:** The impact of capital endowment and various dimensions on the grouping of dietary quality.

Variables	Mild imbalance		Moderate imbalance		Severe imbalance	
	(1)	(2)	(3)	(4)	(5)	(6)
Capital endowment	−72.0590		−17.2347***		−18.0857***	−5.2089**
	(44.1228)		(2.3209)		(3.2220)	
Human capital		−45.1342		−8.6399***		
		(44.5986)		(1.9323)		(2.4005) −12.8771***
Social capital		−44.7421		−6.8625***		
		(37.6721)		(2.6198)		(4.1639) −2.2563
Financial capital		−4.5963		−4.3608***		
		(12.9696)		(1.1442)		(1.8017) −1.3599
Physical capital		18.1073		−5.5757***		
		(24.9924)		(1.7403)		(2.4878)
Control variables	Yes	Yes	Yes	Yes	Yes	Yes
Constant	81.9155	66.9667	61.3313***	64.3837***	61.4483***	62.5228***
	(57.6684)	(55.7356)	(2.9849)	(3.3699)	(4.3566)	(4.8749)
Pseudo R^2^	0.6878	0.7568	0.5971	0.5752	0.7507	0.7287
N	35	35	867	867	354	354

***p < 0.001;

**p < 0.05. The data in parentheses represent the robust standard error.

Low-imbalance group: Neither overall capital endowment nor its individual dimensions show significant effects. However, due to the very small sample size (only 35 observations), these estimates are highly unstable and likely overfitted (pseudo-R^2^ > 0.68). Therefore, no substantive conclusions should be drawn from this subgroup; these results are presented for descriptive purposes only and require validation with larger samples.

Moderate-imbalance group: Overall capital endowment, as well as human, social, financial, and physical capital, all exert significant negative effects on dietary imbalance (all at *p* < 0.001). For the majority of older adults in this “intermediate state,” capital endowment can function through multiple pathways: human capital may enhance health awareness and dietary decision-making; social capital may provide informational support and dietary care; while financial and physical capital directly alleviate economic constraints, promoting food accessibility and diversity.

Severe-imbalance group: Only human capital and social capital remain significant, while financial and physical capital no longer show significant effects. This difference may indicate that nutritional issues in the severe-imbalance group extend beyond economic dimensions, involving deeper noneconomic factors such as cognitive dysfunction, entrenched behavioral habits, or social isolation. Consequently, purely economic support may be insufficient, whereas behavioral changes and social support facilitated by human and social capital become more critical.

Notably, the overall improvement effect of capital endowment is stronger for the severe-imbalance group than for the moderate-imbalance group, suggesting that capital investments may yield higher marginal returns in extreme situations. From a dimensional perspective, human capital plays a more significant role in the moderate-imbalance group, implying greater potential for health education and capacity-building when problems are not yet severe. In contrast, social capital is more prominent in the severe-imbalance group, reflecting its irreplaceable role in alleviating severe social isolation and providing care support.

#### 3.4.2 Heterogeneity across income groups

Household per capita income may moderate the pathways and strength of capital endowment’s effect on dietary imbalance. To examine this heterogeneity, we divided older adults into “below-average income” (*n* = 873) and “above-average income” (*n* = 383) groups based on the median household per capita income.

Table 8 presents the grouped regression results. Overall capital endowment significantly improves dietary imbalance in both income groups (coefficients significant at the 1% level), but the dimensions and mechanisms differ systematically.

**TABLE 8:** Impact of capital endowment on dietary quality in different income groups.

Variables	below-average income		above-average income	
	(1)	(2)	(3)	(4)
Capital endowment	−17.5457***		−15.5992***	
	(1.9190)		(6.0631)	
Human capital		−5.8933***		−15.3240***
		(1.6749)		(3.9286)
Social capital		−8.5403***		−6.6491
		(2.2970)		(5.8443)
Financial capital		−4.1858***		−6.3796***
		(1.0209)		(2.1876)
Physical capital		−6.9408***		−1.5693
		(1.5609)		(3.4445)
Control variables	Yes	Yes	Yes	Yes
Constant	62.0960***	64.5122***	62.3006***	68.4855***
	(2.6481)	(2.9785)	(7.9504)	(9.0145)
Pseudo R^2^	0.6881	0.6707	0.5936	0.5805
N	873	873	383	383

***p < 0.001. The data in parentheses represent the robust standard error.

Below-average income group: All four dimensions of capital endowment—human, social, financial, and physical capital—exhibit significant negative effects (all at *p* < 0.001), indicating that multidimensional capital collectively forms a safety net against nutritional risks. For this group, physical capital may directly improve food access, while social capital provides support through informal mechanisms (e.g., meal assistance from relatives and friends, community mutual aid), demonstrating the “multidimensional complementarity” of capital endowment under resource constraints.

Above-average income group: Only human capital and financial capital remain significant, while social and physical capital no longer show significant effects. This may be because higher-income households have surpassed basic resource constraints, reducing the marginal contribution of material conditions to dietary quality. Additionally, their social networks may serve non-nutritional purposes (e.g., information acquisition, social participation) rather than direct food support.

Further comparison of coefficient magnitudes reveals that the effects of human and financial capital are stronger in the higher-income group, suggesting that education-enhanced health literacy and income-supported purchasing power can be more efficiently translated into dietary quality improvements in a resource-abundant environment.

These findings reveal the income-stratified characteristics of capital endowment’s mechanisms: for low-income older adults, the “safety net” function of social and physical capital should be emphasized; for higher-income groups, the “enhancement” effects of human and financial capital should be prioritized. Therefore, policy interventions must precisely match capital types to the economic characteristics of target groups to maximize the efficiency of nutritional improvement.

## 4 Discussion and implication

### 4.1 Discussion

This study elucidates the multidimensional mechanisms through which capital endowment affects dietary quality among rural elderly in Heilongjiang Province. Theoretically, capital endowment can be deconstructed into four dimensions—human, financial, social, and physical—which operate through complementary and synergistic effects to collectively shift dietary patterns from merely meeting basic survival needs toward pursuing dietary balance. Several findings warrant in-depth discussion.

First, human capital is identified as the most dynamic and core element, primarily operating through the formation of healthy eating concepts and the acquisition of scientific nutritional knowledge. Older households with higher human capital are more capable of interpreting nutritional information, mastering food pairing techniques, and translating such knowledge into reasonable dietary practices. This finding aligns with health demand theory and human capital theory (23) and underscores the importance of nutrition education and health literacy interventions.

Second, the U-shaped relationship for financial capital is a novel and counterintuitive finding. At low levels, increased income helps alleviate undernutrition—a finding consistent with conventional wisdom. However, the right-ascending arm of the U-curve suggests that beyond a certain threshold, additional financial resources may actually worsen dietary imbalance. We propose three complementary explanations:

(a) Shift in consumption motivation: As capital accumulates, dietary motivation evolves from “subsistence-oriented” to “health-oriented” and further to “hedonic consumption” (15). This transition may lead to increased dining out and consumption of highly processed, sugar-rich, or fatty foods.
(b) Precautionary saving motives: rural elderly facing income uncertainty and lacking robust social safety nets (particularly for healthcare) may channel additional cash income into precautionary savings rather than immediately translating it into higher-quality food consumption (17). This “rainy day” saving behavior, while rational from an individual risk-management perspective, can dampen the expected nutritional return on increased financial capital.
(c) Persistence of long-formed dietary habits: Consumption decisions among older adults are deeply influenced by long-formed dietary preferences, lifestyle habits, and risk-averse psychology (24). Even with relaxed budget constraints, they may still allocate additional income to non-food domains or opt for saving rather than purchasing higher-priced healthy foods.

This finding challenges linear policy approaches that assume income transfers alone will improve nutrition and highlights the importance of synergistic interactions with human capital (knowledge conversion), social capital (behavioral modeling and resource expansion), and physical capital (feasibility assurance).

Third, the inverted U-shaped relationship for physical capital reflects its dual role. In the early accumulation phase, investing in major assets (e.g., housing renovations, agricultural equipment) may crowd out daily food expenditures, worsening dietary imbalance (16, 19). Only after assets generate sustainable returns (e.g., rental income, reduced labor burden) can they contribute to better nutrition. The inverted U-shaped impact essentially reflects the role transition of physical capital from a “consumptive burden” to a “productive tool”. That is, the early accumulation stage requires sacrificing current consumption (including dietary consumption), while the later stage allows capital returns to supplement daily consumption.

Fourth, heterogeneity results have important implications for targeting. The finding that only human and social capital matter for the severely malnourished group suggests that purely economic interventions will be ineffective for this subgroup. Instead, home-based nutrition education and community mutual aid networks are needed. Similarly, the differential patterns across income groups indicate that low-income households require a “safety net” approach addressing all capital types, while higher-income households benefit most from human and financial capital enhancements. This finding carries important policy implications: nutrition intervention programs targeting rural elderly should not be limited to economic measures such as income subsidies. Instead, comprehensive strategies are needed, including synchronous efforts to strengthen nutrition education, improve community food environments, provide age-appropriate food storage facilities, and leverage the demonstrative and supportive functions of social networks.

### 4.2 Implications

#### 4.2.1 Policy implications

Based on our findings, we propose the following context-sensitive policy recommendations. Cautionary note: These implications are derived from rural Heilongjiang and should be validated before generalization to other regions.

(a) Prioritize human capital interventions. Given that human capital has the strongest and most consistent effects across subgroups, nutrition education programs for rural elderly should be expanded. These could include community-based workshops on dietary guidelines, easy-to-understand informational materials, and cooking classes tailored to elderly needs. Special attention should be paid to the severely malnourished subgroup, where human capital interventions may yield the highest marginal returns.
(b) Design financial interventions to avoid the U-shaped trap. Simple cash transfers may backfire beyond a certain threshold. Conditional cash transfers (e.g., subsidies specifically for fruits, vegetables, or high-quality protein) or voucher programs could help channel additional income toward healthy foods rather than hedonic consumption. This is particularly important for higher-income older adults who may be at risk of shifting toward “enjoyment-oriented” dietary patterns.
(c) Leverage social capital for severe malnutrition cases. For the 28% of respondents with severe dietary imbalance, social capital interventions (e.g., community mutual aid networks, meal assistance programs, regular home visits by village health workers) may be more effective than economic support alone. Given that social capital remains significant in the severe-imbalance group while financial capital does not, policies should prioritize social inclusion and community support for this vulnerable subgroup.
(d) Recognize the dual role of physical capital. Policies promoting asset accumulation (e.g., housing improvement programs, agricultural equipment subsidies) should be paired with short-term nutritional support to prevent crowding-out effects during the accumulation phase. This is particularly relevant for low-income households where physical capital accumulation may temporarily worsen dietary quality.
(e) Stratify interventions by income and imbalance severity. One-size-fits-all policies are likely inefficient. Low-income households need integrated, multidimensional support that addresses all capital types simultaneously. Higher-income households benefit more from targeted education and healthy food subsidies that leverage their existing human and financial capital. For severely malnourished older adults, interventions should prioritize social and human capital over purely economic measures.

#### 4.2.2 Limitations and future research

This study has several limitations that should be acknowledged.

Geographic generalizability: The data are limited to rural Heilongjiang Province (2022). China’s vast geography encompasses substantial regional variation in dietary culture, economic development, and social structure. As a northeastern province with distinct dietary patterns (e.g., high grain and meat consumption), Heilongjiang’s results may not fully represent southern, southwestern, or coastal rural regions. Our findings should be generalized to other rural regions with caution and validated through replication studies.

Measurement limitations: The 3-day 24-hour dietary recall method is subject to recall bias, which may be more pronounced among elderly populations, potentially leading to systematic underreporting of snacks, condiments, or between-meal consumption. The short recording period (July-August) fails to capture seasonal, festive, or temporary dietary adjustments, which may be particularly relevant in agricultural regions with harvest cycles.

Conceptual simplification: Our quantitative indicators for social capital (e.g., number of relatives, cadre experience, gift money received) do not fully capture qualitative dimensions such as trust, reciprocity, or emotional support. Similarly, the DBI, while valid for overall imbalance assessment, may mask specific nutrient-level deficiencies (e.g., protein, iron, calcium, vitamin D). Different capital types may have differential effects on specific nutrients, which warrants future investigation.

Causal inference: Most importantly, the cross-sectional nature of our data precludes strong causal claims. While we have theoretically discussed and empirically tested for endogeneity using IV methods, the possibility of reverse causality or omitted variable bias cannot be entirely ruled out. For example, the observed positive association between human capital and dietary quality could partly reflect that individuals who are inherently more health-conscious are both more likely to adopt healthy diets and to invest in their own health knowledge.

Future research directions: Future studies should (a) expand geographic coverage to multiple provinces (e.g., southern, southwestern, coastal regions) to test external validity and identify regional variations; (b) establish longitudinal cohorts to establish temporal order and causality; (c) develop age-appropriate dietary assessment tools that minimize recall bias and capture seasonal variations; (d) investigate nutrient-specific effects of different capital types (e.g., protein, iron, calcium) using more disaggregated outcome measures; (e) conduct mixed-methods research to capture qualitative dimensions of social capital (trust, reciprocity, emotional support) that quantitative indices may miss.

## 5 Conclusion

Based on survey data from rural Heilongjiang Province (2022) and employing Tobit models with instrumental variable robustness checks, this study analyzes the impact of capital endowment on dietary quality among rural elderly. The following conclusions are drawn.

First, capital endowment significantly improves dietary quality, but effects vary by dimension. Capital endowment effectively suppresses dietary imbalance, alleviates insufficient intake, and reduces excessive intake. Human capital has the strongest impact, followed by social and physical capital, with financial capital having a relatively weaker effect. Higher capital levels are associated with reduced dietary imbalance, lower excessive intake, and reduced insufficient intake. This ordering—human capital having the largest effect—suggests that the efficiency of converting resources into healthy diets, driven by knowledge and cognitive abilities, is paramount.

Second, the relationship is nonlinear, with dimension-specific patterns. Financial capital exhibits a U-shaped relationship with dietary imbalance: initial improvements followed by deterioration after a threshold. This reflects the evolution of dietary motivation from “survival-oriented” to “health-oriented” to “hedonic consumption.” Physical capital exhibits an inverted U-shaped relationship: initial worsening followed by eventual improvement. This reflects the dual role of assets—as a “consumptive burden” in the early accumulation phase and as a “productive tool” in the later phase when capital returns can supplement daily consumption. No significant nonlinear patterns were detected for human or social capital.

Third, effects are heterogeneous across subgroups. For moderately imbalanced diets, all four capital types contribute significantly. For severe imbalance, only human and social capital remain effective, suggesting that severe malnutrition involves noneconomic factors (e.g., cognitive decline, social isolation) that require human and social capital interventions. Low-income groups rely on a multidimensional safety net of all capital types, while high-income groups are primarily influenced by human and financial capital.

These findings reject linear, one-dimensional approaches to nutritional policy and support tailored, multidimensional interventions that match capital types to the specific constraints of target populations. The results contribute to the literature by (a) identifying U-shaped and inverted U-shaped patterns for financial and physical capital respectively, (b) revealing systematic heterogeneity across income and malnutrition severity subgroups, and (c) demonstrating the primacy of human capital in improving dietary quality among rural elderly. These insights offer crucial implications for promoting healthy aging and nutritional equity in rural China.

## Data Availability

The original contributions presented in this study are included in this article/Supplementary material, further inquiries can be directed to the corresponding author.
