# Supplementary material for: How does capital endowment affect dietary quality in rural elderly? Evidence from Heilongjiang Province, China
Source: Front Nutr. 2026 Jul 14;13:1816331. doi: 10.3389/fnut.2026.1816331 (PMC13407291; doi:10.3389/fnut.2026.1816331)
Supplement: Supplementary file 1 [file Table_1.docx]

Supplemental Table S1. Average Marginal Effects of Capital Endowment on Dietary Quality

|  | (1) | (2) | (3) |
| --- | --- | --- | --- |
|  | DQD | HBS | LBS |
| Panel A. Capital Endowment (Table 3) |  |  |  |
| Capital Endowment | -11.6995*** | -2.4418** | -4.9785** |
| Tobit β | (-13.7639) | (-6.1046) | (-7.6593) |
| Pseudo R² | 0.3564 | 0.0340 | 0.1809 |
| Panel B. Capital Dimensions (Table 4) |  |  |  |
| Human Capital | -6.3219*** | -6.2953*** | -3.3290** |
| Tobit β | (-7.4375) | (-12.5905) | (-5.1215) |
| Social Capital | -6.1876** | -1.0612 | -3.3521 |
| Tobit β | (-7.2795) | (-2.1224) | (-5.1571) |
| Financial Capital | -3.2170*** | -1.3996 | -0.6406 |
| Tobit β | (-3.7847) | (-2.7992) | (-0.9855) |
| Physical Capital | -5.3604* | -3.0074* | -3.7105*** |
| Tobit β | (-6.3063) | (-6.0147) | (-5.7084) |
| Pseudo R² | 0.3624 | 0.0375 | 0.2129 |
| Control variables | Yes | Yes | Yes |
| Region effect | Yes | Yes | Yes |
| Observations | 1256 | 1256 | 1256 |

Note: *** p < 0.001, ** p < 0.05, * p < 0.10. AMEs are reported in bold; Tobit coefficients in parentheses. See Section 3.1 for details.
